# Identification of Candidate Genes and Functional Pathways Associated with Body Size Traits in Chinese Holstein Cattle Based on GWAS Analysis

**DOI:** 10.3390/ani13060992

**Published:** 2023-03-08

**Authors:** Ismail Mohamed Abdalla, Jiang Hui, Mudasir Nazar, Abdelaziz Adam Idriss Arbab, Tianle Xu, Shaima Mohamed Nasr Abdu, Yongjiang Mao, Zhangping Yang, Xubin Lu

**Affiliations:** 1 College of Animal Science and Technology, Yangzhou University, Yangzhou 225009, China; 2 Joint International Research Laboratory of Agriculture and Agri-Product Safety, Yangzhou University, Yangzhou 225009, China

**Keywords:** Chinese Holstein, body size, GWAS, FarmCPU, SNP, functional analysis

## Abstract

**Simple Summary:**

Body size is a significant economic trait for dairy cows due to its significant influence on the production, health, selection, and environmental adaptation of dairy cattle. However, measurements of body size are widely used to predict body weight. Managers of dairy farms also use them to assess the development and growth of the animals during the rearing process. Therefore, understanding the genetic basis of inter-individual variation of body size might accelerate future efforts aimed at dairy cattle improvement. Our genome-wide association study identified several genes and functional pathways associated with body size traits in Chinese Holstein cattle. Results of this study might serve as a foundation for genetic improvement programs for dairy cattle that are based on molecular genetics.

**Abstract:**

Body size is one of the most economically important traits of dairy cattle, as it is significantly associated with cow longevity, production, health, fertility, and environmental adaptation. The identification and application of genetic variants using a novel genetic approach, such as genome-wide association studies (GWASs), may give more insights into the genetic architecture of complex traits. The identification of genes, single nucleotide polymorphisms (SNPs), and pathways associated with the body size traits may offer a contribution to genomic selection and long-term planning for selection in dairy cows. In this study, we performed GWAS analysis to identify the genetic markers and genes associated with four body size traits (body height, body depth, chest width, and angularity) in 1000 Chinese Holstein cows. We performed SNPs genotyping in 1000 individuals, based on the GeneSeek Genomic Profiler Bovine 100 K. In total, we identified 11 significant SNPs in association with body size traits at the threshold of Bonferroni correction (5.90 × 10^−7^) using the fixed and random model circulating probability unification (FarmCPU) model. Several genes within 200 kb distances (upstream or downstream) of the significant SNPs were identified as candidate genes, including *MYH15*, *KHDRBS3*, *AIP*, *DCC*, *SQOR*, and *UBAP1L*. Moreover, genes within 200 kb of the identified SNPs were significantly enriched (*p* ≤ 0.05) in 25 Gene Ontology terms and five Kyoto Encyclopedia of Genes and Genomes pathways. We anticipate that these results provide a foundation for understanding the genetic architecture of body size traits. They will also contribute to breeding programs and genomic selection work on Chinese Holstein cattle.

## 1. Introduction

Body size in humans, cattle, and other domestic animals has been widely investigated [1,2,3,4]. Body size traits were commonly used as the primary breeding selection criterion to monitor cattle growth and to evaluate the selection response [5,6,7]. The profitability of dairy production is largely determined by cows’ ability to produce a large amount of milk. This is in addition to other factors, such as health, fertility, and feed efficiency, as well as management practices. Body size measurements have been used as predictors of body weight in dairy cattle [8]. Hip height and heart girth are the essential traits widely accepted as the most satisfactory method to predict body weight because they are more easily obtained than body weight [9]. Over the last years, heart girth and hip height measurements have been routinely collected from birth to first calving in Chinese Holstein cattle. Heritability estimates for these traits are moderate to high (0.33–0.40) [5]. There have been reports of positive genetic correlations of body weight with milk and protein yield [10]. Furthermore, body weight has an impact on dairy cow health and fertility. For example, calving ease and calf survival are moderately correlated with cows’ body weight and calves’ birth weight [11]. The interval between calving and first service was shorter in heavier cows, but conception rates decreased as body weight increased [12]. In contrast, the improved non-return rates after 56 and 90 days was associated with increasing the heifers’ body weight; body depth was genetically correlated with many other economic traits, such as calving interval (0.35), the days from calving to first insemination (0.79), and gestation period (0.34) [10]. Similarly, gestation period has been shown to have a genetic correlation with body height (0.49) [13]. Interestingly, numerous studies have demonstrated that cows with higher body height have shorter longevity [8,14]. Furthermore, good depth of heart girth in cattle indicates good forage convertibility and feet and leg conformation [15]. On the other hand, studying the genetic basis of body size variation among individuals might help understand the mechanism of environmental adaptation of cattle [7].

For over a decade, GWAS has become an effective approach for detecting genetic markers that are associated with multiple economic traits in animal production. Some GWASs on body size have been carried out across different populations in order to understand the genetic mechanisms of growth traits, such as Chinese Holstein cattle [5], Simmental beef cattle in China [6], and Brahman and Yunling cattle [7]. In addition, various SNPs, genes, and QTLs were related to body measurement, body weight, and conformation traits in different breeds [16,17,18,19]. Therefore, our study aims to identify SNPs associated with body size traits (body height, body depth, chest width, and angularity) using the GWAS approach. Furthermore, we conducted gene ontology and KEGG pathway analysis for a better understanding of the biological functions of the genes within the significant SNPs. The newly identified genetic markers and candidate genes may contribute to genomic selection and the genetic improvement of body size in Chinese Holstein dairy cows.

## 2. Materials and Methods

### 2.1. Ethical Statement

The entire procedures involving animal care, collecting samples of hair follicles, and measurement of phenotypic traits were performed in strict compliance with the guidelines provided by the China Council on Animal Care and the Agricultural Ministry, China. This research was also accepted by the Institutional Animal Care and Use Committee of School of the Yangzhou University Animal Experiments Ethics Committee (License Number: SYXK (Su) IACUC 2020-0910), Yangzhou University. During the collection of samples and data, no animals were uncomfortable or malnourished.

### 2.2. Phenotypic Data and DNA Samples Collection

Four farms located in the province Jiangsu, China, were used to select 1000 Chinese Holstein cows for the experiment. Body height (BH), body depth (BD), chest width (CW), and angularity (ANG) were measured by three trained technicians. They were recorded on a point scale from 1 to 9 in accordance with the China National Standard Code of the practice of type classification in Chinese Holstein (GB/T 35568-2017). In addition, 50 hair follicle samples were obtained from each cow for genotypic analysis.

### 2.3. Phenotypic and Genetic Parameters

The computer-based software IBM-SPSS, (Version 25.0. Armonk, NY, USA: IBM Corp.), was used to estimate the pairwise Pearson correlation coefficients and to determine the descriptive statistic of phenotypic traits (BH, BD, CW, and ANG).

Genetic correlations and heritability among the four traits of body size were estimated using the DMU software (derivative-free approach to multivariate analysis) [20] with the animal model, as below.

$$Y_{ijklm}=u+Herd_{I}+Year_{j}+Season_{k}+Parity_{l}+a_{m}+e_{ijklm}\;$$

where *Y_ijklm_* is the phenotype in the *j*th year, *k*th season, and *l*th parity of the *m*th individual from *i*th herd; *u* is overall mean of the population, *Herd_i_* is the herd effect according to a cow’s origin from one of the four herds; *Year_j_* is the *j*th year effect, *Season_k_* is the *k*th season effect, and $Parity_{l}$ is the effect of *l*th parity; *a* is the additive effect of *m*th individual, and e is the residual in the *j*th year, *k*th season, and lth parity of the *m*th individual from *i*th herd. All effects were treated as fixed except the additive effect. There are at least three generations of pedigree data (age) available for the cows (2009–2020), their parities range from 1 to 4, and four seasons during the measurement time were June–August, September–November, December–February, and March–May [21,22].

### 2.4. Genotyping and Quality Control

Hair follicle samples have been used to extract genomic DNA. DNA extraction and genotyping was carried out using the GeneSeek Genomic Profiler Bovine 100 K SNP Chip at Neogen Biotechnology (Shanghai, China) Co., Ltd. ((http://www.neogenchina.com.cn), accessed on 28 June 2020), based on ARC-UCD1.2/bosTau9 as the genome reference. Quality control was performed using Plink 1.90 software [23] to exclude SNPs: (1) the SNPs with a call rate less than 90%; (2) all SNPs with a MAF (minor allele frequency) less than 0.05; (3) those that violated the HWE (Hardy Weinberg equilibrium) value (*p* < 1.0 × 10^−6^). A total of 984 cows and 84,406 SNPs were kept for subsequent analyses following quality control.

### 2.5. Population Stratification

GWAS can be confounded by population stratification. Therefore, if not properly corrected population stratification can produce spurious associations. Our previous work [22] revealed that the first two principal components (PCAs) had been fitted as covariate variables in the association analysis to eliminate the influence of population stratification.

### 2.6. Genome-Wide Association Studies

The GWAS was performed using the fixed and random model circulation probability unification (Farm-CPU) model [24]. Iteratively, The FarmCPU method uses a fixed effect model (FEM) and a random effect model (REM). Using Plink software, v1.90 [23], the SNP genotypes coded for the association analyses were converted to 0, 1, and 2. The SNPs that passed the significant threshold in the FEM were detected as pseudo quantitative trait nucleotides (QTNs). The pseudo QTNs were subsequently verified using the REM, where the kinships were built using alternative sets of pseudo QTNs. Iterative calculations were carried out through the FEM and the REM until no updated pseudo QTNs exceeded the significance threshold. False positive correlations are mainly caused by population stratification [25]. Therefore, the FEM tests SNPs one at a time. Two of the highest PCAs, accounting for 21% of the population stratification in the FEM, were considered as covariates in order to account for the other genetic variations, except for the pseudo QTNs [26]; the FEM can be given as:

(1) $$y={\;\mathrm{Xb}}_{x}+M_{t}b_{t\;}+{\;S}_{j}d_{i}+e$$

where y is the vector of the adjusted phenotypic values for BH, BD, CW, or ANG traits; $b_{X}$ is the corresponding effect of the first two PCAs, and X is the corresponding coefficient matrix; $b_{t}$ is the fixed effect of the tth pseudo QTN, which was detected by the FEM and optimized by the REM in each cycle, and $M_{t}$ is the corresponding genotype matrix; $S_{j}$ is the genotype of the jth marker, which was converted to 0, 1, or 2, and $d_{j}$ is the effect of the jth marker; and e is the random residual of the model.

Markers have their own *p*-value after substitution. The *p*-values and the associated marker map are used to update the selection of pseudo QTNs using the SUPER algorithm (Settlement of MLM Under Progressively Exclusive Relationship) [27] in a REM, as follows:

(2) y= u+e

where y is the vector of the adjusted phenotypic values of BH, BD, CW, or ANG; u is the vector of total genetic effects of individuals and is assumed to satisfy $u=\left(0,\;K\sigma_{u}^{2}\right)$, in which K is the kinship matrix constructed by the QTNs obtained from the FEM, and $\sigma_{u}^{2}$ is the unknown genetic variance; and e is the random residual of the model.

The false positive associations (type 1 error) rate was set at 5%. The *p*-value (5.9 × 10^−7^) of the significance for SNPs was determined based on the Bonferroni correction method (0.05/N) [28], where N is total SNPs number left after quality control.

### 2.7. Gene Identification and Functional Analysis

The UCSC genome browser via an Asian server for cow assembly (April 2018) (UCSC Genome Browser Gateway) (accessed on 29 May 2022) and the full National Center for Biotechnology Information gene (NCBI) database (National Center for Biotechnology Information (nih.gov) (accessed on 29 May 2022) were used to identify genomic regions and candidate genes. The linkage disequilibrium (LD) analysis performed in [22] identified genes within the 200 kb region of the significant SNPs as candidate genes.

We submitted the GWAS candidate genes to the Database for Annotation, Visualization, and Integrated Discovery (DAVID) [29] for the Gene Ontology (GO) terms and Kyoto Encyclopedia of Genes and Genomes (KEGG) pathway analysis. For functional and pathway analysis, a significant *p* value was set at *p* ≤ 0.05. The online Search Tool for the Retrieval of Interacting Genes (STRING) database, v11.0 [30], was used to investigate protein–protein interactions (PPI) between genes, and the resulting PPI network was visualized using Cytoscape software (v3.8.2).

## 3. Results

### 3.1. Descriptive Statistics and Genetic Parameters Estimation of Body Size Traits

The descriptive statistics of phenotypic measurements of body size traits (BH, BD, CW, ANG), including mean, maximum, and minimum values, are shown in Table 1, where the mean of measurements of these traits ranges between 6.18 and 8.2 scale scores. Genetic and phenotypic correlations between body size traits are given in Table 2. The genetic correlations between these traits revealed highly positive correlations between BH, BD, and CW. At the same time, ANG had a moderate positive correlation with BH and a low positive correlation with other traits. In contrast, the pairwise estimation of phenotypic correlation was high between BD and CW, while BH had low phenotypic correlations with BD and CW. Heritability, as well as genetic and phenotypic correlations between body size traits, are presented in Table 2. The heritability estimation results for BH, BD, CW, and ANG were 0.48, 0.1, 0.17, and 0.19, respectively, as shown in Table 2.

### 3.2. Candidate Genes Association

**Figure 1:**
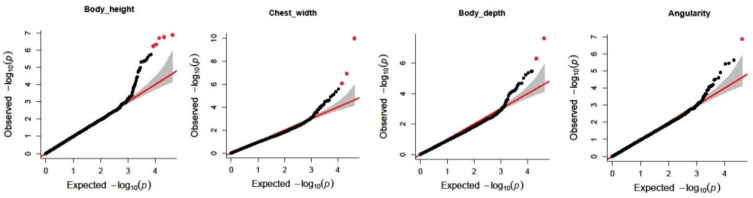
Quantile–quantile plots of GWAS for body size traits in Chinese Holstein cattle. The red lines (expected values) illustrate the null hypothesis of no true association. The observed values (black dots) are a deviation from this line. The grey shades illustrate the confidence interval of the *p*-value. The red dots represent the significant SNPs of the body size traits.

**Figure 2:**
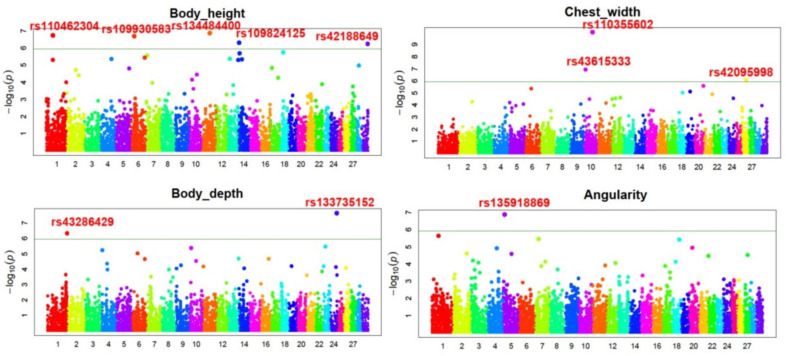
Manhattan plots of GWAS for body size traits in Chinese Holstein cattle. The *p* values (−log10) are plotted against their respective positions on each chromosome. After Bonferroni correction, the green line illustrates the significance level of the threshold.

The GWAS analyses were performed based on the Farm-CPU model. Quantile–quantile plots suggested no inflation or clear systematic bias in the study (Figure 1). Because the model used was well controlled for the population stratification, indicating that the inflation factor (λ) at each trait is near 1 (0.9 < 1.01), a slight deviation in the upper right tail from the diagonal line indicates the significant SNPs associated with the target traits. Manhattan plots (Figure 2) are used to illustrate the significance level of SNPs of GWAS results based on chromosome location. Here, each dot corresponds to a single SNP. The *x*-axis represents genomic position, while the *y*-axis displays the negative of the log *p* value.

The results of the GWAS analyses identified 11 significant SNPs related to body size conformation traits in Chinese Holstein cattle, which statistically passed the threshold (s 5.9 × 10^−7^) after Bonferroni correction, as shown in Table 3.

Five SNPs were associated with BH trait, including the SNP rs134484400 on Chr. 11, the SNP rs110462304 on Chr.1, which is positioned within myosin heavy chain 15 gene (*MYH15*), the SNP rs109930583 on Chr. 6 is located within *LOC112447047* gene and chromosome 6 C4orf17 homolog gene (*C6H4orf17*), the SNP rs109824125 on Chr. 14 is close (100 kb) to KH RNA binding domain containing gene (*KHDRBS3*), and the SNP rs42188649 on Chr. 29 is located within aryl hydrocarbon receptor interacting protein gene (*AIP*). Two SNPs were associated with BD trait; the SNP rs133735152 on Chr. 24 is located near (100 kb) to the DCC netrin 1 receptor gene (*DCC*), and the SNP rs43286429 on Chr. 1 is located close (100 kb) to the *LOC112447004* gene. Moreover, three SNPs were associated with CW; the SNP rs110355602 on Chr. 10 is positioned close (200 kb) to sulfide quinone oxidoreductase gene (*SQOR*), the SNP rs43615333 on Chr. 10 is located within ubiquitin associated protein 1 like gene (*UBAP1L*), and SNP rs42095998 on Chr. 26. is positioned within vesicle transport through interaction with t-SNAREs 1A gene (*VTI1A*). In addition, one SNP was related to the ANG trait; the SNP rs135918869 on Chr. 5 is located close (100 kb) to the coiled-coil domain, containing 59 gene (*CCDC59*) (Table 3).

### 3.3. Gene-Set Enrichment and Analysis

**Figure 3:**
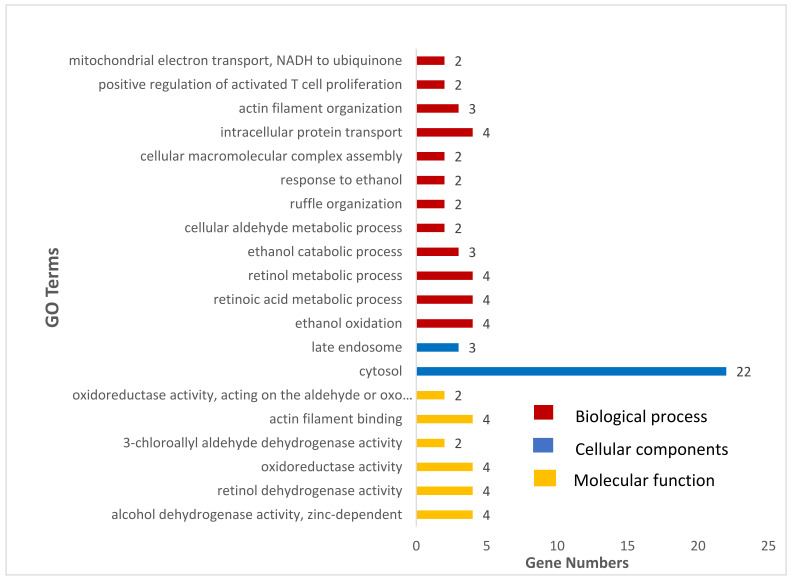
Gene Ontology terms enriched by genes associated with body size.

To gain a deeper understanding of the biological functions shared by the trait-associated genes, we analyzed 105 genes (Table S1) within the region of 200 kb (up/downstream) of the significant SNPs for the four traits of body size. We then conducted KEGG and GO enrichment analysis. Gene ontology enrichment analysis (Table S2 and Figure 3) revealed six biological process terms (GO:0004745:retinol dehydrogenase activity, GO:0016491:oxidoreductase activity, GO:0004024:alcohol dehydrogenase activity, zinc-dependent, GO:0004028:3-chloroallyl aldehyde dehydrogenase activity, GO:0051015:actin filament binding, and GO:0016620:oxidoreductase activity, acting on the aldehyde or oxo group of donors, NAD, or NADP as acceptor), two cellular component terms (GO:0005829:cytosol and GO:0005770~late endosome), and 12 molecular function terms (GO:0006069:ethanol oxidation, GO:0042573:retinoic acid metabolic process, GO:0042572:retinol metabolic process, GO:0006068:ethanol catabolic process, GO:0006081:cellular aldehyde metabolic process, GO:0031529:ruffle organization, GO:0045471:response to ethanol, GO:0034622:cellular macromolecular complex assembly, GO:0006886:intracellular protein transport, GO:0007015:actin filament organization, GO:0042104:positive regulation of activated T cell proliferation, and GO:0006120:mitochondrial electron transport, NADH to ubiquinone). Furthermore, the KEGG pathways (Table 4), which were significantly over-represented (*p* ≤ 0.05) by the set of genes, may have been associated with body size involving 10 pathways (bta00980:Metabolism of xenobiotics by cytochrome P450, bta05204:Chemical carcinogenesis, bta00982:Drug metabolism-cytochrome P450, bta00350:Tyrosine metabolism, bta00010:Glycolysis/Gluconeogenesis, bta00071:Fatty acid degradation, bta00340:Histidine metabolism, bta00410:beta-Alanine metabolism, bta01100:Metabolic pathways, and bta00830:Retinol metabolism).

**Figure 4:**
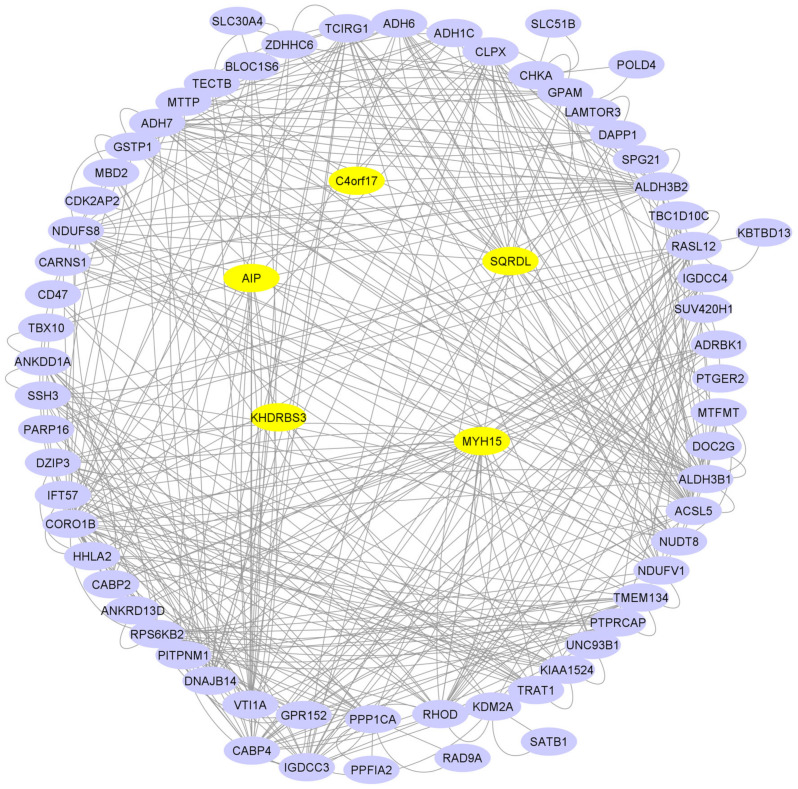
Protein–protein interactions between genes associated with body size traits in Chinese Holstein. (In the yellow nodes, significant candidate genes and their interactions are illustrated).

A PPI analysis was conducted using the STRING database for all the genes previously analyzed as part of the functional analysis. Figure 4 shows a number of significant interactions between genes (77 nodes are connected by 217 edges).

## 4. Discussion

Body size plays a crucial role in dairy cattle’s production, health, selection, and environmental adaptation. However, body size measurements are widely used to predict body weight. In addition, they are commonly used by dairy farmers to monitor growth and track the development of their animals’ developmental progress during the rearing period. Therefore, mapping and detection of significant SNPs and candidate genes influencing body size traits have high economic benefits for dairy cattle breeding. In the current work, we identified 11 SNPs and their associated candidate genes (Table 3), which significantly correlated with the four body size traits (BH, BD, CW, and ANG); five of these significant SNPs were related to the BH trait. BH is one of the quantitative traits that has long fascinated geneticists and is usually highly heritable; understanding the molecular basis of inter-individual variation in this trait might provide novel insights into the mechanisms controlling individual growth [31]. Among these SNPs, the rs110462304 SNP on Chr. 1 has been previously reported in a QTL region associated with carcass trait performance [32]. In our study, this SNP was located within the *MYH15* gene, which belongs to the myosin heavy chain gene family. Myosins are superfamily genes of eukaryotic motor proteins that bind actin and use ATP hydrolysis energy to contribute significantly to a wide range of biological processes, such as muscle contraction, cytokinesis, cell motility and contractility, and intracellular trafficking [33]. A previous meta-analysis of GWAS revealed that the *MYH15* gene is listed as a plausible candidate gene for detecting pleiotropic polymorphisms for body height, reproduction, and fatness in beef cattle [34], as well as pleiotropic effects on body composition in sheep [35]. Over the last few years, the *MYH15* gene has been reported in several studies in chickens, such as RNA sequencing results, which validated that the *MYH15* gene was associated with muscle structures [36], as well as comparative transcriptome analysis reporting that this gene is one of the genes involved in breast muscle growth, regulation, and development in three breeds of chicken [37]. The *MYH15* gene was also related to skeletal muscle myoblast proliferation and differentiation among layer and broiler chickens [38]. The SNP rs109824125 is on Chr. 14 is significantly associated with BH, which has been previously found in some QTLs related to fat milk percentage and milk solids percentage for Thai dairy cattle [39]. This SNP is located near to *KHDRBS3* gene, which is a member of the signal transduction and the activator of RNA (STAR) family proteins. It has been reported that this gene is associated with the 305-day milk yield trait in two cattle breeds: Girolando crossbreed cattle [40] and primiparous Holstein cows [41]. Gene network interactions analysis, derived from GWAS-based results, revealed this gene is related to meat quality and growth traits of Brazilian Nelore beef cattle [42], as well as carcass weight trait in Hanwoo cattle [43]. Deng et al. [44] reported that this gene is one of the suggested candidate genes that might play a role in milk production traits in buffaloes. The rs42188649 was another significant SNP related to BH trait, which is located within AIP gene; this gene has been found to be evolutionarily conserved among species and is widely expressed throughout the organism [45,46]. Recent studies provided insight into the body size variation in cetaceans, indicating that this gene was related to tall stature and overgrowth [47], and a genome-wide scan for selection signatures revealed that this gene was related to cardiac structure and function in Atlantic killifish [48]. Another multi-breed GWASs on dual-purpose bovine behavior traits identified this gene as related to rumination [49].

In this study, SNP rs133735152 was significantly associated with the BD trait, which is close to the *DCC* gene. Several studies, including that of Sunirmal Sheet et al. [50], identified this gene as a candidate gene associated with obesity-related traits in canines based on GWAS analysis. In addition, genome-wide analysis of copy number variations identified the *DCC* gene to be probably associated with morphological, milk, meat, healthy, and reproductive traits in three indigenous Iranian river buffaloes [51].

The most significantly associated SNP in the present analysis (*p* = 9.45 × 10^−11^), rs110355602, was located on Chr. 10, which is associated with BD trait; the rs110355602 SNP is located close to the *SQOR* gene. The *SQOR* is a mitochondrial inner membrane-coding gene in humans that plays an essential role in catalyzing and metabolizing hydrogen sulfide (H2S.) [52]. Similarly, the *SQOR* gene has been also associated with the growth and muscle development traits in Chinese Simmental beef cattle [53]. On the same chromosome (Chr. 10), we found another SNP (rs43615333), which is located within the *UBAP1L* gene; this gene was also significantly associated with BD trait in our results.

Various metabolic activities occur during an animal’s body growth and development, so it is reasonable that the metabolic pathways are one of the significantly enriched KEGG pathways in this study. In the present work, many GO terms (Table S2 and Figure 3) and KEGG pathways (Table 4) were significantly enriched (*p* ≤ 0.05). Among them, five pathways (metabolic pathways, fatty acid degradation, drug metabolism-cytochrome P450, metabolism of xenobiotics by cytochrome P450, and chemical carcinogenesis) were reported to be associated with residual feed intake in Australian Angus cattle [54]. Moreover, retinol metabolism was significantly over-represented in biological pathways involved in carcass trait performance in Holstein-Friesian cattle [32]. In contrast, histidine metabolism was significantly enriched in pathways related to mammary teat-shape conformation traits in Chinese Holstein cattle [55]. In addition, tyrosine metabolism and beta-alanine metabolism were identified as significant KEGG pathways of the differentially expressed genes in the Large White pigs [56].

Besides the *ALDH3B1* gene, three members of ADHs genes (*ADH1C*, *ADH7*, and *ADH6* gene) were displayed in the top six significantly enriched GO terms (zinc-dependent, alcohol dehydrogenase activity, ethanol oxidation, retinol dehydrogenase activity, retinoic acid metabolic process, retinol metabolic process, and ethanol catabolic process). Moreover, these genes are also revealed in most KEGG pathways in the current study. Zhao et al. [57] reported that *ADH6* in three enriched KEEG pathways (bta00830:Retinol metabolism, bta00982:Drug metabolism-cytochrome P450, and bta00980:Metabolism of xenobiotics by cytochrome P450) of the functional analysis of differentially expressed genes in the down and up group are based on transcriptome analysis of ruminal epithelia related to the gradual high fermentable dietary transition in beef cattle. The *ADH1C* gene has an interactive effect with vitamin A supplementation level on intramuscular fat content in beef steers [58], while Ghafouri et al. [59] reported the *ADH1C* gene to be downregulated in the lipolysis process in poultry based on the integration of RNA-Seq and microarray data approach. The *ADH1C* gene also has been reported in another study using text mining technique to identify genes associated with meat quality and carcass traits in Hanwoo cattle [60]. In their study, Wang et al. [61] found that this gene was linked with hot carcass weight, carcass marbling, lean meat yield, and rib eye area in beef cattle. The authors of this study showed that a direct association and biological function of this gene are related to small molecule biochemistry and lipid metabolism. Interestingly, this gene also has been involved in lipid metabolism, and small molecule biochemistry biological functions for daily dry matter intake in a study integrated plasma metabolites and imputed whole genome sequence variants in beef cattle [62]. This gene was also differentially expressed in beef steers’ livers for average daily gain and daily dry matter intake in the Charolais breed [63]. Rafael Medeiros de Oliveira et al. also identified the *ALDH3B1* gene in a GWAS work for backfat thickness in Nellore cattle [64].

There is some interest in the fact that the most significant genes in this study were related to growth and body development traits of beef cattle in several previous studies, which might be attributed to the importance of body size as one of the main breeding selection criteria in beef cattle breeding.

## 5. Conclusions

In summary, we identified 11 significant SNPs associated with four body size traits (BH, BD, CW, and ANG) using FarmCPU-based GWAS in Chinese Holstein cattle. This study revealed the six most promising candidate genes (*MYH15*, *KHDRBS3, AIP, DCC, SQOR*, and *UBAP1L*); in addition, five KEGG pathways and 25 GO terms were significantly enriched. These results provide novel insights into the molecular breeding basis and highlight useful information for understanding the genetic architecture of body size traits in dairy cattle. Thus, they contributed to the genomic selection of Chinese Holstein cows. These findings offer valuable information. However, further studies will be required to investigate the biological functions and the molecular regulatory network of the candidate genes.

## Figures and Tables

**Table 1:** Descriptive statistics of body size traits of Chinese Holstein cows (1000).

Trait	Mean	Std. Error	Min.	Max.	Std. Deviation
BH	7.8608	0.04402	1.00	9.00	1.3807
BD	7.1189	0.05837	3.00	9.00	1.8308
CW	8.2053	0.03573	4.00	9.00	1.1206
ANG	6.1753	0.03895	2.00	9.00	1.2218

BH, body height; BD, body depth; CW, chest width; ANG, angularity.

**Table 2:** Genetic correlations (upper diagonal), pairwise Pearson phenotypic correlations (lower diagonal), and heritability estimations (grey diagonal line) of body size traits.

Trait	BH	BD	CW	ANG
BH	0.48 (0.03)	0.939	0.703	0.517
BD	0.096	0.10 (0.01)	0.8578	0.052
CW	0.164	0.784	0.17 (0.01)	0.173
ANG	0.140	−0.334	−0.277	0.19 (0.01)

BH, body height; BD, body depth; CW, chest width; ANG, angularity.

**Table 3:** Information related to identified SNPs associated with body size traits.

Trait	SNP	rs. SNP	Chr.	Position (bp)	MAF	*p-*Value	Nearest Gene	Distance (kb)
BH	BovineHD1100016691	rs134484400	11	57,877,493	0.3748	1.36 × 10^−07^	-	-
	ARS-BFGL-NGS-18743	rs110462304	1	53,214,714	0.3391	1.86 × 10^−07^	*MYH15*	Within
	Hapmap28262-BTA-143868	rs109930583	6	25,113,885	0.0643	2.06 × 10^−07^	*LOC112447047* *C6H4orf17*	Within
	Hapmap23799-BTC-047701	rs109824125	14	6,680,908	0.3411	4.98 × 10^−07^	*KHDRBS3*	100 kb
	ARS-BFGL-NGS-24800	rs42188649	29	45,369,368	0.1940	5.80 × 10^−07^	*AIP*	Within
BD	BovineHD2400015228	rs133735152	24	53,151,923	0.4279	2.33 × 10^−08^	*DCC*	100 kb
	BTB-00074122	rs43286429	1	155,166,581	0.2502	4.71 × 10^−07^	*LOC112447004*	100 kb
CW	BovineHD1000018705	rs110355602	10	64,626,909	0.3784	9.45 × 10^−11^	*SQOR*	200 kb
	BovineHD1000004064	rs43615333	10	12,056,720	0.4366	1.17 × 10^−07^	*UBAP1L*	Within
	BTB-00939179	rs42095998	26	33,060,404	0.2293	8.22 × 10^−07^	*VTI1A*	Within
ANG	BovineHD0500003481	rs135918869	5	11,715,037	0.1649	1.32 × 10^−07^	*CCDC59*	100 kb

BH, body height; BD, body depth; CW, chest width; ANG, angularity; SNP, single nucleotide polymorphisms; Chr., chromosome; Position, position (bp) on ARC-UCD1.2; MAF, minor allele frequency; Distance (kb), the distance between identified SNPs and the nearest gene; Nearest Gene, nearest genes found on the UCSC Genome browser and NCBI database (ARC-UCD1.2/bosTau9), *p* Value; *p*-values calculated from the Farm-CPU model analysis.

**Table 4:** KEGG pathways enriched by the candidate genes of genome-wide significant SNPs associated with body size traits.

Terms	Description	%	*p*-Value	Gene Name
bta00982	Drug metabolism-cytochrome P450	0.063892	1.49 × 10^−07^	*LOC508879*, *ADH1C*, *GSTP1*, *ALDH3B1*, *ADH7*, *ADH6*
bta00980	Metabolism of xenobiotics by cytochrome P450	0.063892	1.65 × 10^−07^	*LOC508879*, *ADH1C*, *GSTP1*, *ALDH3B1*, *ADH7*, *ADH6*
bta05204	Chemical carcinogenesis	0.063892	4.65 × 10^−07^	*LOC508879*, *ADH1C*, *GSTP1*, *ALDH3B1*, *ADH7*, *ADH6*
bta00350	Tyrosine metabolism	0.045637	2.47 × 10^−05^	*LOC508879*, *ADH1C*, *ALDH3B1*, *ADH7*, *ADH6*
bta00010	Glycolysis/Gluconeogenesis	0.045637	1.50 × 10^−04^	*LOC508879*, *ADH1C*, *ALDH3B1*, *ADH7*, *ADH6*
bta00071	Fatty acid degradation	0.03651	7.24 × 10^−04^	*ADH1C*, *ACSL5*, *ADH7*, *ADH6*
bta00340	Histidine metabolism	0.027382	0.004052	*LOC508879*, *ALDH3B1*, *CARNS1*
bta00410	beta-Alanine metabolism	0.027382	0.009524	*LOC508879*, *ALDH3B1*, *CARNS1*
bta01100	Metabolic pathways	0.109529	0.012732	*POLD4*, *NDUFS8*, *LOC508879*, *GPAM*, *CHKA*, *ADH1C*, *ALDH3B1*, *ACSL5*, *TCIRG1*, *ADH7*, *NDUFV1*, *ADH6*
bta00830	Retinol metabolism	0.027382	0.024631	*ADH1C*, *ADH7*, *ADH6*

## Data Availability

The data presented in this study are available upon request from the corresponding author.

## Supplementary Materials

The following supporting information can be downloaded at: https://www.mdpi.com/article/10.3390/ani13060992/s1, Table S1: Genome-wide significant SNP associated with body size traits and genes (within 200 kb distances, upstream or downstream).; Table S2: Gene ontology terms for body size traits were significantly enriched (*p* ≤ 0.05).
