# Supplementary material for: Identification of Candidate Genes and Functional Pathways Associated with Body Size Traits in Chinese Holstein Cattle Based on GWAS Analysis
Source: Animals (Basel). 2023 Mar 8;13(6):992. doi: 10.3390/ani13060992 (PMC10044097; doi:10.3390/ani13060992)
Supplement: Supplementary file 1 [file animals-13-00992-s001.zip › Table S1.pdf]

Table S1. Genome-wide significant SNP associated with body size traits and genes (within 200 kb distances, upstream or downstream).

Traits	SNP name	Chr.	Position	*p* value	Gene name
Body height	BovineHD1100016691	11	57877493	1.36E^{-07}	-
	ARS-BFGL-NGS-18743	1	53214714	1.86E^{-07}	*MYH15*<br>*HHLA2*<br>*CIP2A*<br>*DZIP3*<br>*CD47*<br>*IFT57*<br>*CIP2A*<br>*DZIP3*<br>*TRAT1*<br>*LOC112447297*
	Hapmap28262-BTA-143868	6	25113885	2.06E^{-07}	*LOC112447047*<br>*C6H4orf17*<br>*DNAJB14*<br>*LAMTOR3*<br>*DAPP1*<br>*MTTP*<br>*TRMT10A*<br>*C6H4orf17*<br>*ADH1C*<br>*ADH6*<br>*LOC101903868*<br>*DAPP1*<br>*C6H4orf54*<br>*ADH7*<br>*LOC781689*
	Hapmap23799-BTC-047701	14	6680908	4.98E^{-07}	*KHDRBS3*<br>*LOC112449600*<br>*LOC101903327*<br>*LOC112449601*
	ARS-BFGL-NGS-24800	29	45369368	5.80E^{-07}	*AIP*<br>*SYT12*<br>*RHOD*<br>*GRK2*<br>*ANKRD13D*<br>*SSH3*<br>*POLD4*<br>*RAD9A*<br>*PPP1CA*<br>*TBC1D10C*<br>*RPS6KB2*<br>*PTPRCAP*<br>*CORO1B*<br>*CABP4*

					TMEM134<br>*CDK2AP2*<br>*CABP2*<br>*GSTP1*<br>*NDUFV1*<br>*DOC2G*<br>*NUDT8*<br>*UNC93B1*<br>*ALDH3B1*<br>*NDUFS8*<br>*TCIRG1*<br>*KMT5B*<br>*KDM2A*<br>*GRK2*<br>*ANKRD13D*<br>*SSH3*<br>*LOC107131975*<br>*CLCF1*<br>*RAD9A*<br>*TBC1D10C*<br>*CARNS1*<br>*LOC112444920*<br>*CARNS1*<br>*RPS6KB2*<br>*LOC112444921*<br>*GPR152*<br>*PITPNM1*<br>*CDK2AP2*<br>*CABP2*<br>*GSTP1*<br>*LOC112444865*<br>*NDUFV1*<br>*DOC2G*<br>*NUDT8*<br>*TBX10*<br>*LOC508879*<br>*UNC93B1*<br>*LOC112444853*<br>*ALDH3B1*<br>*NDUFS8*<br>*TCIRG1*<br>*CHKA*
Body depth	BovineHD2400015228	24	53151923	2.33E^{-08}	*DCC*<br>*MBD2*
	BTB-00074122	1	155166581	4.71E^{-07}	*LOC112447004*<br>*SATB1*

					LOC112448269<br>*LOC101908237*<br>*LOC112448155*
Chest width	BovineHD1000018705	10	64626909	$9.45E^{-11}$	*SQOR*<br>*SLC30A4*<br>*LOC101902552*<br>*BLOC1S6*<br>*LOC112448495*
	BovineHD1000004064	10	12056720	$1.17E^{-07}$	*UBAP1L*<br>*PTGER2*<br>*SPG21*<br>*MTFMT*<br>*SLC51B*<br>*RASL12*<br>*PDCD7*<br>*PARP16*<br>*ANKDD1A*<br>*SLC51B*<br>*KBTBD13*<br>*CLPX*<br>*LOC112448368*<br>*PARP16*<br>*LOC112448638*<br>*LOC112448637*<br>*IGDCC3*
	BTB-00939179	26	33060404	$8.22E^{-07}$	*VTI1A*<br>*GPAM*<br>*TECTB*<br>*ACSL5*<br>*ZDHHC6*<br>*LOC112444522*<br>*ZDHHC6*<br>*MIR11981*<br>*TECTB*<br>*MIR6715*
Angularity	BovineHD0500003481	5	11715037	$1.32E^{-07}$	*CCDC59*<br>*PPFIA2*<br>*METTL25*<br>*LOC104972346*
